# One-year randomized trial comparing virtual reality-assisted therapy to cognitive–behavioral therapy for patients with treatment-resistant schizophrenia

**DOI:** 10.1038/s41537-021-00139-2

**Published:** 2021-02-12

**Authors:** Laura Dellazizzo, Stéphane Potvin, Kingsada Phraxayavong, Alexandre Dumais

**Affiliations:** 1 Research center of the Institut Universitaire en Santé Mentale de Montréal, Montreal, Canada; 2 Department of Psychiatry and addictology, Faculty of Medicine, University of Montreal, Montreal, Canada; 3 Services et Recherches Psychiatriques AD, Montreal, Canada; 4 Institut national de psychiatrie légale Philippe-Pinel, Montreal, Canada

**Keywords:** Schizophrenia, Psychosis

## Abstract

The gold-standard cognitive–behavioral therapy (CBT) for psychosis offers at best modest effects. With advances in technology, virtual reality (VR) therapies for auditory verbal hallucinations (AVH), such as AVATAR therapy (AT) and VR-assisted therapy (VRT), are amid a new wave of relational approaches that may heighten effects. Prior trials have shown greater effects of these therapies on AVH up to a 24-week follow-up. However, no trial has compared them to a recommended active treatment with a 1-year follow-up. We performed a pilot randomized comparative trial evaluating the short- and long-term efficacy of VRT over CBT for patients with treatment-resistant schizophrenia. Patients were randomized to VRT (*n* = 37) or CBT (*n* = 37). Clinical assessments were administered before and after each intervention and at follow-up periods up to 12 months. Between and within-group changes in psychiatric symptoms were assessed using linear mixed-effects models. Short-term findings showed that both interventions produced significant improvements in AVH severity and depressive symptoms. Although results did not show a statistically significant superiority of VRT over CBT for AVH, VRT did achieve larger effects particularly on overall AVH (*d* = 1.080 for VRT and *d* = 0.555 for CBT). Furthermore, results suggested a superiority of VRT over CBT on affective symptoms. VRT also showed significant results on persecutory beliefs and quality of life. Effects were maintained up to the 1-year follow-up. VRT highlights the future of patient-tailored approaches that may show benefits over generic CBT for voices. A fully powered single-blind randomized controlled trial comparing VRT to CBT is underway.

## Introduction

Schizophrenia is among the top medical disorders that produce disability worldwide^1^. Among the core symptoms of the illness, auditory verbal hallucinations (AVH), which are the experience of hearing the voice of a person or of an entity that is not present physically, are the most commonly reported form of hallucinations with a lifetime prevalence of 70% in this population^2^. AVH can have a devastating effect on a patient’s life due to high levels of distress^3^, feelings of depression^4^, impaired social functioning^5^, increased suicide risk^6^, and delayed recovery^7^. The felt distress is mainly due to the negative/derogative content of voices (i.e., threatening, frightening, hostile voices)^8,9^. Unfortunately, not all patients respond to antipsychotic medication. It has been estimated that 20–50% of patients will have treatment-resistant schizophrenia (TRS)^10–13^. Moreover, only 30–60% of these patients will respond to clozapine^14–16^. TRS is associated with some of the highest levels of impaired functioning^17^, rates of hospitalization^18^, and costs to society^19^. Thus, schizophrenia, especially TRS, is a complex, severe, and disabling psychiatric disorder that poses significant therapeutic challenges^20^.

With the recognition of the limitations, side effects and health risks associated with antipsychotic medication^21,22^, psychosocial interventions have become extensively endorsed in clinical practice guidelines as part of the treatment of those with psychotic experiences^23,24^. The most widely studied evidence-based and first-line psychological treatment recommended from guidelines for psychotic symptoms is cognitive–behavioral therapy for psychosis (CBTp)^25–27^, which comprises an umbrella of interventions. The main instrument of change in cognitive–behavioral approaches involves discussing the origins of hallucinations, reframing appraisals, and modifying behavior related to psychotic symptoms, increasing the use of better coping strategies (i.e., mindfulness), reducing distress, and improving well-being^28–30^. The effect sizes across meta-analyses have varied generally depending on the (1) specific population chosen (e.g., poor treatment responders^31^), (2) type of therapy (e.g., case formulation-based^32^), (3) intensity of therapy (e.g., low-intensity^33^), (4) assessed time-points (e.g., post-therapy^34^, follow-up improvements^35^), or (5) comparison groups (e.g., active control^36^). Globally, most studies have found CBTp to be at best moderately effective in ameliorating psychotic symptoms and improving domains of well-being; effects, however, appear weaker when compared with other psychotherapies and at follow-ups^31–33,35–37^. CBTp remains further shortcoming with up to 50% of patients not responding to this approach^38^.

Given these modest treatment effects, and the limited impact of CBTp on AVH, researchers have suggested that the development of interventions should be guided by research on processes specific to the experience of voice-hearing^39^. There is consequently an increasing tendency for CBTp to focus less on changing faulty thinking and to begin to employ supplementary therapeutic methods to highlight ways of relating to the self, emotion regulation, and interpersonal relationships^40^. As highlighted in a systematic review by Lincoln and Peters^41^, these approaches have been shown to yield better effects compared to generic CBTp. Amid these individually tailored interventions are a new wave of relational approaches building on the perspective that AVH are experienced as coming from entities that have personal identities, and with whom the voice-hearer establishes a personal relationship^42–47^. Dialogical therapies (i.e., refs. ^45–50^) aim to ameliorate the voice-hearer’s relationship by encouraging assertive interactions with voices, by negotiating new ways of relating and by ameliorating self-views^51–54^. Different techniques (i.e., role-play with the therapist, empty-chair work) have been used to allow patients to engage with their voices. With advances in technology, AVATAR Therapy (AT) uses a visual depiction of the AVH that enables the therapist to role-play the voice to aid the voice-hearer practice different responses to their experience in a more direct manner^47,55,56^. We have independently extended the therapy using immersive virtual reality (VR) with a head-mounted display to deliver the therapy (VR-assisted therapy (VRT))^46^. The exposure to an avatar of patients’ personified voice is likely to be a unique and robust device to reduce fear and distress associated with persecutory voices, which is to a certain degree similar to exposure-based therapies^41,57,58^. Importantly, this novel intervention enables voice-hearers to converse with their voice in the aims of improving coping and diminishing felt distress by addressing power and control within these relationships as well as by modifying negative self-perceptions and ways of relating^46,55,56^.

The results of the two pilot trials comparing AT/VRT to treatment-as-usual^46,47^ as well as a larger RCT comparing AT to supportive counseling^45^ showed large effects of VR therapies on AVH in short-term follow-ups and up to a 24-week follow-up. The therapy has also shown improvements for overall symptoms of schizophrenia, depressive symptoms, voice malevolence as well as omnipotence, and quality of life. Improvements appeared to be larger than those of conventional treatments. Though, to date, no randomized trial has compared this relational VR therapy to a recommended active treatment with a long-term 12-month follow-up. The aim of this pilot comparative trial was therefore to evaluate the efficacy of VRT over our CBT for AVH adapted for patients with TRS in the short-term and to examine if effects are maintained in time. The trial additionally had for aim to assess the acceptability and feasibility of both interventions and estimate the amplitude (e.g., effect size) of the potential difference in efficacy between both interventions for future larger trials.

## Results

### Sample characteristics

**Fig. 1:**
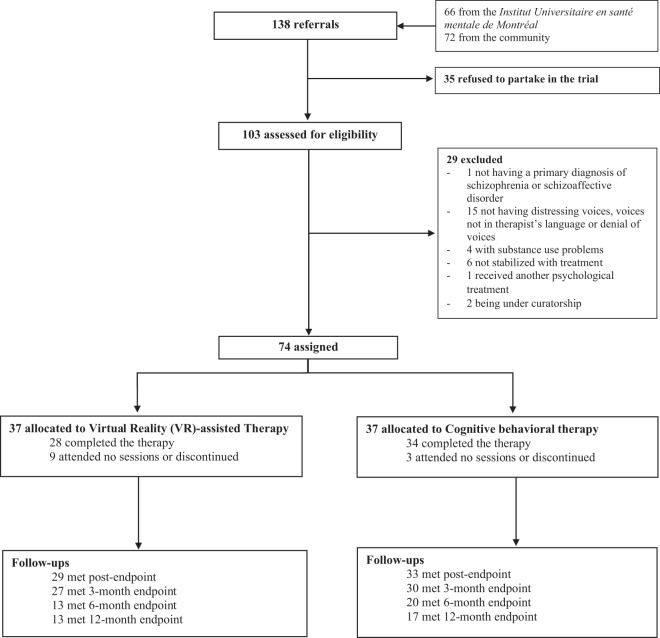
Trial profile of patients who received virtual reality (VR)-assisted therapy or cognitive–behavioral therapy. There were 138 referrals provided by clinical teams and the community, of whom 74 were eligible and randomized to either one of the therapies.

During the study period, 138 referrals were provided by clinical teams and the community, 35 individuals refused to partake in the project, thereby leaving 103 participants that were assessed, of whom 74 were eligible. Reasons for participant exclusion were: not having a primary diagnosis of schizophrenia or schizoaffective disorder (*n* = 1), not hearing distressing voices, reporting voices not speaking in the therapists’ primary language or in denial of voices (*n* = 15), having substance use problems (*n* = 4), not stabilized with treatment (*n* = 6), having received another psychological treatment at the time of the intervention (*n* = 1), and being under curatorship (*n* = 2). Eligible participants were randomized to either VRT (*n* = 37) or CBT for AVH (*n* = 37). For a flowchart of the study participants, please see Fig. 1. Overall, there was a greater proportion of men (76%), most were Caucasian (82%), the mean age was 42.5 years (SD = 12.7), ~80% were single and the mean duration of schooling was 12.2 years (SD = 3.6) (see Table 1 for more details). Most patients held a diagnosis of schizophrenia (77%) with a mean duration of illness of 16 years (SD = 10.4) and were treated with atypical antipsychotics (96%). Over half of participants were also prescribed clozapine but continued to experience persistent AVHs, thereby meeting the criteria for ultra-resistance. At baseline, there were no significant differences between the two groups (VRT and CBT) in terms of psychiatric symptoms and sociodemographic data (*p* > 0.05).

**Table 1:** Baseline sociodemographic and clinical characteristics.

Characteristics	Virtual reality (VR)-assisted therapy	Cognitive–behavioral therapy	Total
	*N* = 37	*N* = 37	*N* = 74
Age (years)	43.6 ± 12.0	41.4 ± 13.4	42.5 ± 12.7
Sex			
Male	78.4%	73.0%	75.7%
Female	21.6%	27.0%	24.3%
Civil status			
Single	83.8%	78.4%	81.1%
Divorced/separated	13.5%	10.8%	12.2%
Married/common in law	2.7%	10.8%	6.8%
Ethnicity			
Caucasian	83.8%	80.6%	82.2%
Visible minorities	16.2%	19.4%	17.8%
Duration of schooling (years)	12.6 ± 3.8	11.9 ± 3.4	12.2 ± 3.6
Primary diagnosis			
Schizophrenia	78.4%	75.7%	77.0%
Schizoaffective disorder	21.6%	24.3%	23.0%
Duration of illness (years)	18.0 ± 10.6	14.6 ± 10.2	16.0 ± 10.4
Medication: antipsychotics			
Atypical	94.6%	97.2%	95.9%
Clozapine	40.5%	55.6%	52.1%

Data are presented as mean ± standard deviation or %.

There were no significant differences between treatment groups.

### Short-term treatment efficacy

As observed in Table 2, several statistically significant within-group improvements were found for VRT and CBT from baseline to 3-month follow-up.

**Table 2:** Outcomes at baseline, post-treatment, and 3-month follow-up for short-term efficacy comparison.

Treatment condition	Mean and standard deviation (SD)						*p*-value	Timepoint comparisons			|Effect size T1–T3|	Time × Treatment interaction
	Baseline		Post-therapy		Three-month follow-up			T2–T1	T3–T1	T3–T2		
	Mean	SD	Mean	SD	Mean	SD		*p*-value	*p*-value	*p*-value		*p*-value
PSYRATS-AH-Total												
VRT	29.592	4.313	23.069	9.410	22.159	8.728	**<0.001**	**<0.001**	**<0.001**	1.000	1.080	0.266
CBT	29.351	6.853	25.549	9.327	24.903	9.026	**0.001**	**0.024**	**0.006**	1.000	0.555	
PSYRATS-AH-Distress												
VRT	15.403	3.248	11.457	5.341	11.111	5.139	**<0.001**	**<0.001**	**<0.001**	1.000	0.998	0.214
CBT	14.264	4.915	12.046	4.982	11.767	6.490	**0.004**	**0.028**	**0.011**	1.000	0.434	
PSYRATS-AH-Frequency												
VRT	6.694	1.864	5.483	2.681	5.222	2.309	**0.005**	**0.035**	**0.021**	1.000	0.701	0.738
CBT	7.446	2.198	6.424	2.818	6.633	2.580	**0.009**	**0.013**	0.178	1.000	0.339	
PSYRATS-AH-Attribution												
VRT	5.444	1.594	4.414	2.338	4.148	2.248	**0.004**	**0.040**	**0.004**	1.000	0.665	0.851
CBT	5.919	1.785	5.091	2.241	4.900	1.900	**0.017**	0.070	**0.028**	1.000	0.553	
PSYRATS-AH-Loudness												
VRT	2.083	0.996	1.724	1.066	1.741	1.095	0.197	0.347	0.403	1.000	0.327	0.207
CBT	1.811	0.908	2.000	1.090	1.600	0.724	0.176	1.000	0.786	0.202	0.257	
BAVQ-R-Total												
VRT	47.000	10.334	44.706	14.990	42.975	20.128	0.404	0.959	0.611	1.000	0.252	0.794
CBT	49.165	16.051	45.451	14.396	42.274	11.871	0.056	0.198	0.072	1.000	0.488	
BAVQ-R-Persecutory beliefs												
VRT	15.000	6.432	12.071	6.981	11.750	8.294	**0.039**	0.098	0.055	1.000	0.438	0.940
CBT	13.203	7.720	10.909	6.559	10.392	6.985	0.076	0.139	0.125	1.000	0.382	
BAVQ-R-Benevolence												
VRT	2.861	3.818	3.607	4.841	3.500	4.624	0.879	1.000	1.000	1.000	0.151	0.470
CBT	4.595	5.346	4.182	4.883	3.867	4.305	0.446	0.884	0.619	1.000	0.150	
BAVQ-R-Engagement												
VRT	2.528	3.342	4.143	5.324	4.000	5.276	0.213	0.296	0.275	1.000	0.333	0.078
CBT	5.000	5.883	4.273	5.299	4.200	5.261	0.266	0.356	1.000	0.980	0.143	
BAVQ-R-Resistance												
VRT	18.483	6.252	16.704	6.955	15.539	8.329	0.183	0.387	0.222	1.000	0.400	0.417
CBT	17.568	6.954	17.788	6.009	16.781	6.204	0.577	1.000	1.000	0.904	0.119	
BDI-II-Total												
VRT	20.580	10.927	15.969	12.863	14.124	11.455	**0.003**	**0.009**	**0.004**	1.000	0.577	0.889
CBT	18.555	10.941	14.424	9.427	12.677	12.626	**0.003**	**0.028**	**0.002**	0.716	0.498	
BDI-II-Cognitive												
VRT	9.446	5.905	6.483	6.127	5.963	5.893	**<0.001**	**<0.001**	**0.001**	1.000	0.590	0.335
CBT	7.622	6.309	5.818	4.283	4.900	5.202	**0.012**	0.106	**0.009**	0.477	0.471	
BDI-II-Somatic-affective												
VRT	11.130	6.211	9.497	7.493	8.162	6.320	0.076	0.361	0.080	1.000	0.474	0.849
CBT	10.934	6.068	8.606	6.134	7.776	8.164	**0.010**	**0.050**	**0.012**	1.000	0.439	
PANSS-Total												
VRT	78.991	13.889	72.443	14.434	70.074	13.485	**0.008**	**0.045**	**0.015**	1.000	0.651	0.284
CBT	75.743	15.697	73.788	16.443	71.652	16.862	0.554	1.000	0.854	1.000	0.251	
PANSS-Positive												
VRT	13.451	3.999	11.690	3.475	12.407	3.895	0.087	0.084	1.000	0.510	0.264	0.349
CBT	13.892	3.857	13.333	4.601	12.567	4.352	0.267	1.000	0.371	1.000	0.322	
PANSS-Negative												
VRT	15.514	5.541	15.180	5.316	14.630	4.789	0.754	1.000	1.000	1.000	0.171	0.864
CBT	14.216	4.685	14.182	4.640	14.080	4.990	0.971	1.000	1.000	1.000	0.028	
PANSS-Disorganized												
VRT	7.297	2.053	6.966	3.053	6.778	2.342	0.816	1.000	1.000	1.000	0.236	0.611
CBT	7.892	2.998	7.818	3.046	7.417	3.091	0.199	1.000	0.221	0.972	0.156	
PANSS-Excited/Hostility												
VRT	7.541	2.631	6.690	1.854	5.963	1.605	**0.005**	0.268	**0.004**	0.114	0.724	0.109
CBT	6.865	2.188	6.788	2.203	6.400	1.958	0.832	1.000	1.000	1.000	0.224	
PANSS-Anxio-depressive												
VRT	10.189	2.999	9.069	2.5062	8.000	2.557	**<0.001**	**0.019**	**<0.001**	0.113	0.786	**0.025**
CBT	9.108	2.622	8.818	2.579	8.600	2.568	0.703	1.000	1.000	1.000	0.196	
Q-LES-Q-SF												
VRT	47.460	7.050	49.456	7.913	52.222	7.890	**0.001**	0.349	**0.001**	0.127	0.637	0.061
CBT	51.204	6.208	52.7879	7.6107	52.400	7.797	0.519	0.781	1.000	1.000	0.170	

Data are raw mean scores with standard deviation (SD). Linear mixed models with maximum-likelihood estimation were used. Significant differences (*p*-value <0.05) are found in bold.

*VRT* virtual reality (VR)-assisted therapy, *CBT* cognitive–behavioral therapy, *PSYRATS-AH* Psychotic Symptoms Rating Scale–Auditory Hallucinations, *BAVQ-R* Beliefs About Voices Questionnaire-Revised, *BDI-II* Beck Depression Inventory-II, *PANSS* Positive and Negative Symptom Scale, *Q-LES-Q-SF* Quality of Life Enjoyment and Satisfaction Questionnaire-Short From.

Both treatment groups showed significant reductions on the prespecified primary outcome consisting of AVH symptoms assessed with the total PSYRATS-AH score (*p* < 0.001 for VRT and *p* = 0.001 for CBT). Significant reductions were found most prominently for distress related to AVH and voice frequency subscales of the PSYRATS. Based on Cohens’ *d*, the effects of VRT on AVH were large (PSYRATS-AH-Total score *d* = 1.080; PSYRATS-AH-Distress *d* = 0.998; PSYRATS-AH-Frequency *d* = 0.701) and small to moderate for CBT (PSYRATS-AH-Total score *d* = 0.555; PSYRATS-AH-Distress *d* = 0.434; PSYRATS-AH-Frequency *d* = 0.339).

Concerning beliefs about voices measured with the BAVQ-R, VRT showed significant improvements from baseline to 3-month follow-up on persecutory beliefs (*p* = 0.039). Although not statistically significant, CBT showed a trend toward significance on the persecutory beliefs subscale (*p* = 0.076) and total beliefs about voices (*p* = 0.056). Both therapies showed moderate effects on persecutory beliefs about voices (*d* = 0.438 for VRT and *d* = 0.382 for CBT). Depressive symptoms as secondary outcomes measured with the BDI-II also diminished in both treatment groups with effects being of moderate magnitude (*d* = 0.577 for VRT and *d* = 0.498 for CBT). Although not statistically significant for the CBT arm, overall general symptoms as measured with the PANSS significantly diminished (*p* = 0.008) with VRT. Most effects were observed on the excited/hostility subscale (*p* = 0.005) and anxio-depressive subscale (*p* < 0.001). The effect of VRT was of moderate range (*d* = 0.651) for overall symptomatology and was found to be larger for affective symptoms (*d* = 0.724 for excited/hostility symptoms and *d* = 0.786 for anxio-depressive symptoms). In addition, VRT significantly ameliorated quality of life (*p* = 0.001) with an effect of moderate magnitude (*d* = 0.637).

There was one statistically significant between-group Time × Treatment effect for the anxio-depressive subscale of the PANSS, yielding to a superiority of VRT over CBT (*p* = 0.025) (see Table 2).

### Long-term maintenance

As shown in Table 3, results for VRT were maintained in the long-term up to the 1-year follow-up with no statistically significant differences from 3-month follow-up for most outcomes. The only exception comprised the engagement subscales of the BAVQ-R for VRT, which was found to diminish significantly (*p* = 0.002) from 3- to 12-month follow-up and returned to baseline. CBT showed no statistically significant differences in any of the outcomes.

**Table 3:** Outcomes at 3-, 6-, and 12-month follow-up for long-term maintenance comparison.

Treatment condition	Mean and standard deviation (SD)						Timepoint comparisons	Time × Treatment interaction
	Three-month follow-up		Six-month follow-up		Twelve-month follow-up		*p*-value	*p*-value
	Mean	SD	Mean	SD	Mean	SD		
PSYRATS-AH-Total								
VRT	22.159	8.728	21.029	11.908	20.615	12.600	ns	ns
CBT	24.903	9.026	24.240	9.412	23.941	12.204	ns	
PSYRATS-AH-Distress								
VRT	11.111	5.139	11.000	6.822	10.462	7.264	ns	ns
CBT	11.767	6.490	12.350	5.669	11.765	6.815	ns	
PSYRATS-AH-Frequency								
VRT	5.222	2.309	4.692	2.562	4.769	2.743	ns	ns
CBT	6.633	2.580	5.600	2.741	5.941	2.989	ns	
PSYRATS-AH-Attribution								
VRT	4.148	2.248	3.539	2.504	3.615	2.434	ns	ns
CBT	4.900	1.900	4.850	2.601	4.647	2.499	ns	
PSYRATS-AH-Loudness								
VRT	1.741	1.095	1.769	1.301	1.769	1.235	ns	ns
CBT	1.600	0.724	1.400	0.883	1.588	1.004	ns	
BAVQ-R-Total								
VRT	42.975	20.128	46.750	17.263	46.611	18.983	ns	ns
CBT	42.274	11.871	43.619	11.055	39.706	14.443	ns	
BAVQ-R-Persecutory beliefs								
VRT	11.750	8.294	13.167	7.383	11.808	8.760	ns	ns
CBT	10.392	6.985	10.900	6.656	10.529	7.107	ns	
BAVQ-R-Benevolence								
VRT	3.500	4.624	4.333	5.069	3.269	4.196	ns	ns
CBT	3.867	4.305	4.650	5.499	3.882	4.986	ns	
BAVQ-R-Engagement								
VRT	4.000	5.276	3.417	4.719	2.154	2.672	**0.005**	**0.047**
CBT	4.200	5.261	4.900	5.999	4.177	6.002	ns	
BAVQ-R-Resistance								
VRT	15.539	8.329	17.333	8.469	18.385	9.421	ns	ns
CBT	16.781	6.204	16.368	5.974	14.529	8.508	ns	
BDI-II-Total								
VRT	14.124	11.455	17.692	12.579	19.615	15.031	ns	ns
CBT	12.677	12.626	10.258	8.570	15.059	13.953	ns	
BDI-II-Cognitive								
VRT	5.963	5.893	7.692	5.618	9.000	7.572	ns	ns
CBT	4.900	5.202	4.650	4.727	5.706	5.839	ns	
BDI-II-Somatic-affective								
VRT	8.162	6.320	10.000	7.572	10.615	7.848	ns	ns
CBT	7.776	8.164	5.605	6.017	9.353	9.117	ns	
PANSS-Total								
VRT	70.074	13.485	76.769	18.895	73.308	12.854	ns	ns
CBT	71.652	16.862	71.800	18.998	73.333	12.212	ns	
PANSS-Positive								
VRT	12.407	3.895	14.231	5.510	13.615	4.646	ns	ns
CBT	12.567	4.352	12.750	4.689	12.726	3.200	ns	
PANSS-Negative								
VRT	14.630	4.789	14.077	4.890	14.385	3.228	ns	ns
CBT	14.080	4.990	13.250	6.016	14.059	5.178	ns	
PANSS-Disorganized								
VRT	6.778	2.342	7.308	3.225	7.231	2.488	ns	ns
CBT	7.417	3.091	7.150	2.925	7.765	2.488	ns	
PANSS-Excited/Hostility								
VRT	5.963	1.605	6.385	2.256	6.077	1.4979	ns	ns
CBT	6.400	1.958	6.350	1.725	6.000	1.4979	ns	
PANSS-Anxio-depressive								
VRT	8.000	2.557	9.308	2.626	8.769	3.468	ns	ns
CBT	8.600	2.568	8.750	3.024	8.235	3.212	ns	
Q-LES-Q-SF								
VRT	52.222	7.890	51.846	6.479	50.769	7.407	ns	ns
CBT	52.400	7.797	54.031	7.079	54.177	8.798	ns	

Data are raw mean score with standard deviation (SD). Linear mixed models with maximum-likelihood estimation were used. *p*-values are given for significant differences only (*p*-value <0.05, in bold).

*VRT* virtual reality (VR)-assisted therapy, *CBT* cognitive–behavioral therapy, *PSYRATS-AH* Psychotic Symptoms Rating Scale–Auditory Hallucinations, *BAVQ-R* Beliefs About Voices Questionnaire-Revised, *BDI-II* Beck Depression Inventory-II, *PANSS* Positive and Negative Symptom Scale, *Q-LES-Q-SF* Quality of Life Enjoyment and Satisfaction Questionnaire-Short From, *ns* non-significant.

### Acceptability and feasibility of interventions

Of the 74 participants, nine withdrew at some stage from VRT and three from CBT. Reasons for withdrawal included lack of motivation, not wanting to reduce their voices and moving away. In terms of adverse events, no patients were re-hospitalized during the totality of the trial. Attrition rate at post-treatment was 16.2% (Fig. 1). Reasons for discontinuation at follow-ups varied and included patients initially having a maximum follow-up period being set at 3 months, patients being unreachable after several attempts and patients not desiring to further participate in the project. In the 15 patients having participated in semi-structured interviews on their perspectives concerning treatments, most participants found their corresponding intervention (VRT or CBT) to be adequate in content, sequence, dose, tailoring, timing, mode of delivery, and equipment use. One-third of participants did find the dose of interventions (VRT and CBT) to be too short and would have preferred supplementary sessions. Particularly related to VRT, 37.5% voiced the intervention as being stressful at first, which is precisely within the scope of VRT. Once they had overcome the initial exposure to anxiety, they enjoyed their experience and found it to be interesting. In addition, 42.9% of participants in CBT found the homework to be either uninteresting as sessions progressed, not enough to gain awareness or lacking visual aid.

## Discussion

With the rise of VR in psychotherapy to enhance conventional approaches, this randomized comparative trial aimed to compare the efficacy of an innovative treatment using VR (VRT) to our adapted CBT for AVH in patients with TRS. Both therapies of nine weeks were found to be feasible to implement and acceptable to patients with no adverse events being attributed to any of the interventions. Drop-out rates, while slightly larger for VRT, were in similar range to other psychosocial interventions^59^. In addition, both interventions were found to be efficacious and yielded notable improvements in symptomatology for patients with persistent symptoms who have not responded to prior treatments. This is a breakthrough as approximately half of patients were considered ultra-resistant and prescribed clozapine.

Concerning key outcomes, our findings showed that both VRT and CBT reduced overall AVH in the short-term, including associated distress and frequency. Although our findings did not show a statistically significant superiority of VRT over CBT, VRT did achieve larger effects particularly on overall AVH (*d* = 1.080), voice distress (*d* = 0.998), and frequency (*d* = 0.701). These effects are in the same range as those observed in the prior trials on VR therapies for AVH^45–47^, which were of large magnitude as well. Markedly, effects were maintained up to our 1-year follow-up. These findings, while not significant, suggest that VR therapies for voices may potentially achieve greater efficacy on AVH in comparison to the small-to-moderate effects that have been observed in literature on generic CBTp, which is corroborated by the moderate effect of our CBT for AVH (*d* = 0.555). Moreover, CBT is generally not meant to reduce frequency in voices as is the case of AT and VRT, but rather change the beliefs patients have towards their voices^60^. In this trial, CBT only showed a trend towards significance on overall beliefs about voices and persecutory voices, with effects both of moderate range (*d* = 0.488 and *d* = 0.382). Effects may have reached significance in this trial if we had a larger sample size. The effect on persecutory beliefs, which combined malevolence and omnipotence, was significant for VRT and similarly attained a moderate effect (*d* = 0.438). This was in accordance with our initial pilot trial that found significant results on both malevolence and omnipotence^46^. Interestingly, we observed a between-group Time × Treatment effect trend toward significance for engagement with voices. This suggests that patients engaged more with their voices following VRT. Since VRT is an experiential therapy that allows patients to engage with a personified version of their distressing voice by emphasizing on the alteration of their emotional experience, patients may more easily increase engagement. This may also extend outside of the therapy sessions. However, the effect then returned to baseline value at the 1-year follow-up, which suggests that booster sessions may be necessary to maintain effects in time.

Of interest, VRT specifically reduced clinician-ranked overall symptoms of schizophrenia in comparison to CBT. Our results suggested a superiority of VRT over CBT on more affective symptoms (i.e., anxio-depressive symptoms) with effects reaching large magnitude. This finding is not surprising given VRT’s emphasis on enabling patients to experience strong emotions (e.g., anxiety, fear, and anger) during the dialog with their voices and to learn to regulate them. The therapy may therefore help reduce cognitive avoidance of fear-relevant information (i.e., the voice and its content) and reduce anxiety as a result of exposure^61,62^. A usual therapy experience for patients who engage with the approach generally involves some early anxiety followed by a reported sense of relief, achievement, power, and liberation^56,63,64^. It has been speculated that distressing AVH with negative content may directly impact mood, and low mood may in turn make a patient more vulnerable to further AVH^65^. Therefore, focusing on emotional regulation and reductions in distress may influence affective symptoms as observed in both our pilot project and this trial. Through our immersive VRT, affective symptoms may be tackled by enabling patients to learn to better manage their intense emotions and to improve their self-image. Beyond VRT sessions, patients appeared to continue to consolidate their learnings into their daily lives, which may likewise explain the significant improvement observed on subjective quality of life. This finding is important as patients with TRS often have poorer quality of life^66^, which is an indicator of their sense of well-being and satisfaction of their life circumstance^67^. Not only has the treatment of patients with schizophrenia been traditionally focused on symptoms, but many psychotherapies have not targeted quality of life. As observed in our trial, CBT has not clearly shown to improve quality of life^68^. In recent years, subjective quality of life has become a particularly crucial target that should be improved with treatment since enhanced quality of life may bring about recovery in patients^69^.

The trial has implications for the treatment of patients with TRS since it showed that both treatment modalities demonstrated significant improvements, which paralleled with their corresponding therapeutic targets as well as delivery modality. Due to the heterogeneity in patients with schizophrenia, voice-hearers may comprise distinct subtypes that require different forms of treatment^70^. In this sense, CBT may be best for patients who are not ready to be immersed into the emotion-inducing experience of VRT and desire to learn more about their AVH. In addition, CBT is ideal for patients who hear voices with no communicative content as it may appear at the least pointless and potentially harmful to attempt to enter a dialog with an absent agent^71^. Nevertheless, this type of manual-based approach may not be adequate for patients with cognitive deficits, may not be sufficiently individually tailored and may not allow to target relevant factors of their hallucinatory experience (i.e., interpersonal aspects of their experience) and rather emphasizes beliefs about voices. Hence, instead of trying to challenge beliefs about voices and learn to resist voices, VRT, in accordance with “Third-wave therapies,” primarily focuses on how patients relate with their voices by working on improving self-esteem, self-acceptance, and emotion regulation. Within this approach, the patient’s relationship with their voice is fundamentally viewed in the context of their current and previous significant relationships^4,72^. VRT may therefore target a range of therapeutic targets that are relevant to the voice-hearing experience and allows patients to experientially live their experience in a secure therapeutic environment, thereby enabling learnings to be more readily transferred to the real world. Nevertheless, VRT may be too anxiogenic and confrontational at first for some patients, which may explain the slightly higher drop-out rate in comparison to CBT. It also remains to be clarified whether VRT is superior or equivalent to traditional relational approaches (e.g., ref. ^50^) and whether it truly necessitates the use of immersive VR in comparison to using a computerized system as in AT^45,47^.

There are noteworthy limitations to this trial that should nonetheless be acknowledged. The most important limits include the evaluators being non-blinded to treatment allocation during the clinical assessments, small sample size, and single therapist per therapy. First, while most outcomes measured used “self-reported” type assessment, the PANSS used clinical judgment. Thus, the evaluation of clinical changes may have been under- or over-estimated. Though, this is less likely the case since evaluators were trained on a series of videos provided from an external provider to ensure interrater reliability. Second, we observed several trends toward significance (*p* < 0.1), which may have become significant if a larger sample size was obtained. Nevertheless, our results were in similar range to those of a full-powered well-conducted randomized trial on AT^45^. Third, another limitation is the fact that the therapy has been offered only by a skilled therapist with substantial expertise in the psychological treatment of schizophrenia. Apart from CBT for AVH, it is, however, unknown if the efficacy of VRT would remain across therapists as this type of therapy poses delivery challenges (e.g., shifting in real time between communicating as therapist and avatar), ethical considerations (e.g., the therapist must recreate critical and hostile interactions), and training dilemmas (e.g., the therapist should be experienced with this specific population). A further single-blind randomized controlled trial comparing VRT to CBTp, which will be sufficiently powered, is underway to target these limitations (ClinicalTrials.gov Identifier: NCT04054778).

In summary, our pilot comparative trial is the first to compare VRT to evidence-based CBT for the treatment of refractory voices in patients with schizophrenia. We showed that both low-intensity treatment groups are beneficial interventions with effects lasting in time. Nevertheless, VRT yielded to larger effects on AVH and showed additional effects on affective symptoms and quality of life. Keeping in mind that there is no sole effective intervention that is likely to benefit all patients, VRT highlights the future of patient-tailored approaches that integrates several processes (i.e., self-experience, emotion regulation) relevant to potentially improve the effectiveness of generic CBT for voices. Since schizophrenia, mostly TRS, is an extremely complex disorder associated with significant impairments in social and occupational functioning, VRT may have implications for patients’ health and quality of life that are potentially immense. Although this study was not conducted with the aim of further understanding the therapeutic elements of the interventions, future research should aim to understand the components of psychotherapies that leads to efficacy over simply conducting efficacy trials. There is indeed emerging work into understanding the therapeutic components that lead to AT’s and VRT’s large efficacy on AVH and other facets of the illness^63,64^. More research is necessary to establish which components of AT/VRT make it efficacious and to determine which patients may respond better to the intervention. Studies are currently underway to better understand the differences between treatment “responders” versus “non-responders”.

## Methods

### Participants

We were referred 138 patients from the Institut Universitaire en Santé Mentale de Montréal and the community. Briefly, patients were eligible if they were 18 years of age or older, had a diagnosis of either schizophrenia or schizoaffective disorder with persistent AVH and failed to respond to two or more antipsychotic trials. Participants were excluded if they presented a neurological disorder, an unstable and serious physical illness, or a substance use disorder in the past year and if they followed CBTp in the past year. The trial was conducted in accordance with the Declaration of Helsinki and was approved by the institutional ethical committee (CER IPPM 16-17-06). We obtained written informed consent from all patients.

### Design

This is a pilot randomized parallel comparative trial comparing two 9-weekly interventions of 1 h: VRT and CBT for AVH. All patients continued to receive standard psychiatric care (treatment as usual) and agreed to withhold from changing existing medication over the duration of the therapy sessions. Patients fulfilling inclusion criteria were randomly assigned (based on a 1:1 ratio) to either VRT or CBT for AVH. Randomization was determined following the completion of baseline assessments by an external research coordinator. Therapy discontinuation from either group was defined as nonattendance to consecutive sessions and discontinuation decided by patients or recommended by the treating therapist (i.e., participant using substances that interfered with the psychotherapy). This clinical trial has been registered on Clinicaltrials.gov (identifier number: NCT03585127).

### Virtual reality (VR)-assisted therapy

Patients generally underwent 9-weekly sessions consisting of one avatar creation session and eight therapeutic sessions where patients were immersed into the VR setting. Of all patients, most (67%) received nine sessions, whereas the rest received seven sessions. The change in the number of sessions from seven in the pilot trial^46^ to nine was based upon consensus with the treating psychiatrist (AD) and the research team that patients necessitated additional consolidation sessions to achieve better treatment effects. The therapy was delivered by an experienced clinician (AD) who has around 7 years of experience as a psychiatrist. In his clinical practice, he has evaluated and treated over one thousand patients with major psychiatric disorders including schizophrenia^73–82^. The therapy was manualized and assessment of the external validity of the delivery of the intervention was performed by a doctoral student (LD). To do so, a random selection of patient sessions was rated based on a scale developed by the team to assess adherence to the manualised approach.

In the first session, patients underwent a comprehensive assessment of AVH and were requested to create and personalize the face and voice of an avatar best resembling the person or entity believed to be the source of their most distressing voice. This was ensured with the aid of a doctoral student (LD) and the treating psychiatrist (AD). Patients who heard several voices were invited to select the most distressing voice or the most dominant one for the creation of the avatar. Patients were immersed in VR through the Samsung Gear VR head-mounted display or, more recently using more advanced technologies, through an Oculus Rift head-mounted display. The platform that was used allowed to quickly and simply create highly realistic synthetic characters. Unity 3D game engine with custom made assets and Morph3D Character System were used to create idiosyncratic avatars. The voice of the avatar was simulated in real time with a voice transformer (Roland AIRA VT-3). Lip synchronization was performed via SALSA with RandomEyes Unity 3D extension. Patients sat in an adjacent separate room from the therapist, who would converse with patients either through the voice of the avatar or as themselves. The immersive virtual environment consisted of an avatar seen from a first-person perspective standing in a dark room. An inventory of facial expressions was integrated into the platform to use at the therapist’s discretion to enable the avatar to express emotions that patients would easily recognize such as joy, sadness, anger, and fear based on the Facial Action Coding System^83^.

The immersive therapeutic sessions consisted of (1) pre-immersion where the therapist would discuss the preceding week and determine the objective of the therapy session with the patients; (2) immersion where the patient would be immersed in the VR environment and be encouraged to enter in a dialog with their avatar animated in real time by the therapist; and (3) post-immersion where the therapist debriefed the patient and evaluated their feelings of their immersive experience. Sessions 2–4 aimed to confront patients to their hallucinatory experience. The therapist induced a dialog between patients and their avatar with the help of sentences they provided, which were generally abusive, critical, and hostile remarks. Patients were incited to enter a dialog with the avatar to enhance emotional regulation and assertiveness. Session 5 targeted self-esteem, which was supported by enabling the patients to express themselves and to consider their personal qualities. To facilitate this process, a list of qualities presented by the patient’s personal surroundings was introduced in the dialog of the avatar. The interaction of the avatar with the patient became less abusive and more supportive as sessions of VRT progressed. The patient generally became more empowered in the interaction they held with their avatar as the former developed more assertiveness. In the final consolidation sessions, patients had the opportunity to apply what they had previously learned in the experiential sessions and to follow-up on their initial objectives.

### Cognitive–behavioral therapy for auditory verbal hallucinations

The active control condition consisted of nine individual and weekly sessions of 1 h. These sessions were administered in an individual format by a licensed psychologist trained in CBT by Dr. O’Connor, who had trained 35 psychologists throughout his career^84–91^. The CBT program was derived and adapted from current evidence-based treatments for AVH^89^. The therapy was manualized, and a doctoral student (LD) performed the assessment of the external validity of the delivery of the intervention, based on a treatment fidelity grid developed by the research team, on a randomly selected sample. Dr. O’Connor likewise ensured the fidelity to the manual by conducting weekly meetings with the treating psychologist.

The intervention involved a succession of learning modules and suggested task assignments. The first contact with the patient consisted of a history of their voices for goal setting and an introduction to the therapy. Sessions 2 and 3 focused on assessing and learning about hallucinations. With the cognitive model of hallucinations (session 3), the voices were comprehended as triggers rather than beliefs. Patients completed voice journals (assignments), which allowed them to understand and reflect on their positive symptoms and associated triggers. The following sessions focused on metacognition. In the 4th session, patients learned about diverse attributional mechanisms and the session included another voice journal to detect the beliefs that were the cause of their ill-being and, in the 5th and 6th sessions, patients were aided to interpret situations in a better manner with the use of vignettes. In sessions 7 and 8, patients practiced mindfulness exercises, were encouraged to ask for feedback and learned to observe. Session 8, including a last voice journal, allowed patients to put forward alternative explanations to their most common beliefs about their hallucinations. Session 9 led to the end of the intervention and aimed to prevent relapse.

### Clinical assessments

Clinical assessments were administered before and after each intervention and at follow-up periods (3-, 6-, and 12 months) by trained psychiatric nurses. The evaluators had several meetings throughout the trial to ensure that all evaluations were conducted in a thorough and consistent manner.

The predetermined primary outcome consisted of the overall severity of AVH up to our 3-month follow-up, which was re-evaluated at 6 and 12 months to ensure maintenance of effects. AVH were evaluated with the total auditory hallucination subscale score of the *Psychotic Symptoms Rating Scale* (PSYRATS-AH)^92^ that comprises 11 items evaluated by interview (0–44). Since AVH are multidimensional, we further chose to examine the subscales of the PSYRATS-AH consisting of distress, frequency, attribution, and loudness. The psychometric properties of the PSYRATS-AH have shown excellent interrater reliability and good validity^92^.

Secondary outcomes included beliefs about voices, overall psychiatric symptoms, and quality of life. Patients’ beliefs about their voices as well as the manner they cope with them were measured with the Beliefs About Voices Questionnaire-Revised (BAVQ-R)^93^. The Cronbach’s *α* for the total scale has been found to be high (mean *α* = 0.86)^93^. Factor analysis has shown that the BAVQ-R supports four subscales^94^: two subscales relating to beliefs (persecutory beliefs combining omnipotence as well as malevolence components, and benevolence) in addition to two further subscales that measure responses to the voices (resistance and engagement). Depressive symptoms were assessed with the Beck Depression Inventory-II (BDI-II)^95^, which consists of a 21-item self-report inventory. The BDI-II was separated into cognitive and somatic-affective components. This instrument has shown high levels of internal consistency (*α* = 0.90) and test-retest reliability ranging from 0.73 to 0.96^96^. Symptoms of schizophrenia were evaluated with the Positive And Negative Syndrome Scale (PANSS)^97^. Evaluators were trained to administer the latter clinical scale by using a series of gold-standard videotapes and by conducting consensus ratings ensuring interrater reliability. This scale has reported good interrater reliability, appropriate test-retest reliability, and high internal reliability^97,98^. This scale was separated into five symptom clusters^99^: positive symptoms (including hallucinations, delusions, and disorganized thoughts, speech, and behavior), negative symptoms (including lack of motivations and social withdrawal), cognitive symptoms (including memory, language, and attention), hostility and excitement symptoms (including impulse control and violence), and anxio-depressive symptoms (including anxiety and depressive symptoms). Life satisfaction was evaluated with the Quality of Life Enjoyment and Satisfaction Questionnaire-Short Form (Q-LES-Q-SF)^100,101^, which consists of a self-report scale of 14 items. This scale has shown high internal consistency and test-retest reliability^102^.

In addition to drop-out rate and attrition, the perspectives of a sub-sample of patients from each treatment arm were examined to assess the acceptance and feasibility of both interventions. Semi-structured interviews were therefore held with patients based on a set of questions from Feeley and Cossette^103^. Questions were aimed at gaining information regarding patients’ views on several factors of the therapies including content, sequence, dose, setting, mode of delivery, and equipment/material used. Interviews were held until data saturation was achieved (eight VRT and seven CBT). These interviews were recorded and then transcribed.

### Analyses

Statistical analyses were performed with SPSS Statistics for Windows (Version 25, IBM). Descriptive statistics were conducted on baseline data to test for group differences. Potential differences in clinical variables (psychosocial, sociodemographic, and pharmacological) at baseline were verified with chi-square tests in the case of dichotomic data and independent *t*-tests in the case of continuous data. Changes in reported outcomes for short-term efficacy, before and after treatment and at 3-month follow-up, were assessed using a linear mixed-effects model with maximum-likelihood estimations for missing data. The same methodology was conducted to test for maintenance in long-term follow-up from 3 to 12 months. Both between-group and within-group comparisons were verified. Time × Treatment group interaction allowed to indicate whether there was a significant change between VRT and CBT over time. The statistical threshold for significance was set at *p* < 0.05. Effect sizes were categorized as small (0.2), medium (0.5), and large (>0.8) effects^104^.

As for acceptability and feasibility of the therapies, patients’ verbatim from the transcripts were classified into main themes comprising content, sequence, dose, tailoring, timing, mode of delivery, and equipment/material used. Verbatim within each theme was then categorized as being satisfactory for patients, unsatisfactory or missing. Reasons for reduced satisfaction were considered. Frequencies of these categorizations for each theme were then calculated.

### Reporting summary

Further information on research design is available in the Nature Research Reporting Summary linked to this article.

## Supplementary information

Reporting summary

## Data Availability

The data that support the findings of this study are available from the corresponding author upon reasonable request.
